# A model to control the epidemic of H5N1 influenza at the source

**DOI:** 10.1186/1471-2334-7-132

**Published:** 2007-11-13

**Authors:** Y Guan, H Chen, KS Li, S Riley, GM Leung, R Webster, JSM Peiris, KY Yuen

**Affiliations:** 1 State Key Laboratory of Emerging Infectious Diseases, the University of Hong Kong, Pokfulam, Hong Kong SAR, China; 2 Department of Microbiology, the University of Hong Kong. Pokfulam, Hong Kong SAR, China; 3 Research Center of Infection and Immunology, the University of Hong Kong, Pokfulam, Hong Kong SAR, China; 4 International Institute of Infection and Immunity, Shantou University Medical College, Shantou, China; 5 Department of Community Medicine, the University of Hong Kong, Pokfulam, Hong Kong SAR, China; 6 School of Public Health, the University of Hong Kong, Pokfulam, Hong Kong SAR, China; 7 Virology Division, Department of Infectious Diseases, St. Jude Children's Research Hospital, Memphis, TN 38105, USA

## Abstract

**Background:**

No country is fully prepared for a 1918-like pandemic influenza. Averting a pandemic of H5N1 influenza virus depends on the successful control of its endemicity, outbreaks in poultry and occasional spillage into human which carries a case-fatality rate of over 50%. The use of perimetric depopulation and vaccination has failed to halt the spread of the epidemic. Blanket vaccination for all poultry over a large geographical area is difficult. A combination of moratorium, segregation of water fowls from chickens and vaccination have been proved to be effective in the Hong Kong Special Administrative Region (HKSAR) since 2002 despite endemicity and outbreaks in neighbouring regions. Systematic surveillance in southern China showed that ducks and geese are the primary reservoirs which transmit the virus to chickens, minor poultry and even migratory birds.

**Presentation of the hypothesis:**

We hypothesize that this combination of moratorium, poultry segregation and targeted vaccination if successfully adapted to an affected district or province in any geographical region with high endemicity would set an example for the control in other regions.

**Testing the hypothesis:**

A planned one-off moratorium of 3 weeks at the hottest month of the year should decrease the environmental burden as a source of re-infection. Backyard farms will then be re-populated by hatchlings from virus-free chickens and minor poultry only. Targeted immunization of the ducks and geese present only in the industrial farms and also the chickens would be strictly implemented as blanket immunization of all backyard poultry is almost impossible. Freely grazing ducks and geese would not be allowed until neutralizing antibodies of H5 subtype virus is achieved. As a proof of concept, a simple mathematical model with susceptible-infected-recovered (SIR) structure of coupled epidemics between aquatic birds (mainly ducks and geese) and chickens was used to estimate transmissibility within and between these two poultry populations. In the field the hypothesis is tested by prospective surveillance of poultry and immunocompetent patients hospitalized for severe pneumonia for the virus before and after the institution of these measures.

**Implications of the Hypothesis:**

A combination of targeted immunization with the correct vaccine, segregation of poultry species and moratorium of poultry in addition to the present surveillance, biosecurity and hygienic measures at the farm, market and personal levels could be important in the successful control of the H5N1 virus in poultry and human for an extensive geographical region with continuing outbreaks. Alternatively a lesser scale of intervention at the district level can be considered if there is virus detection without evidence of excess poultry deaths since asymptomatic shedding is common in waterfowls.

## Background

The influenza A H5N1 virus has caused diseases in over 200 human and the culling of millions of poultry. Most human cases were recognized to be poultry-to-human transmission [1]. Therefore the control of this virus should be focused on the poultry side. Highly pathogenic influenza A H5N1 virus was first identified in 1959 amongst the chickens of Scotland. The ancestor of current poultry H5N1 strains, Goose/Guangdong/1/96, was subsequently detected during an outbreak of highly pathogenic avian influenza in the poultry of southern China in 1996. A year later in HKSAR, an outbreak of 18 human cases with six deaths was preceded by massive chicken deaths in farms and markets[2]. A series of control measures in HKSAR has successfully controlled the outbreak in human and poultry. In 2003, a HKSAR family returned from Fujian Province with two virologically confirmed cases[3]. This was followed by a major outbreak in the poultry and human of Southeast Asia which still cannot be controlled by perimetric depopulation and vaccination of chickens and is now spreading to Central Asia, Middle East, Africa and also Europe. A consecutive four-year poultry surveillance in South China showed that virus isolation rate started to rise when the ambient temperature was below 20°C[4], and peaked when the temperature went below 10°C. The virus is known to survive at a lower temperature for weeks but less than a week at high ambient temperature of 37°C in faecal materials[5]. Our recent study suggests that the virus had been endemic in poultry for about 10 years [6]. It demonstrated that asymptomatic poultry maintain and amplify the viruses, and also serve as infectious source for other farm poultry and migratory birds, thereby maintaining and disseminating the outbreak. The virus was probably spread by both poultry trafficking and migratory ducks and geese, which tend to interact with domestic ducks and geese. The overall virus isolation rate in asymptomatic market poultry is about 1% but 90% of these isolates came from geese and ducks. Furthermore, monthly breakdown showed that isolation in ducks and geese preceded those of the chicken and minor poultry (Table 1). We hypothesize that the poultry epidemic can be controlled by limiting geese and ducks to in-house industrial farms with vaccination and by a one-off 3 week moratorium of all poultry farming at the hottest month to interrupt the cycle of transmission.

**Table 1:** Number of poultry positive for influenza A H5N1 virus per population sample in one Chinese province over a 12-month period between 2004–2005.

Month	Chicken	Duck	Geese	Minor poultry*
Jul	0/127	0/117	0/60	0/0
Aug	0/147	4/264	0/3	0/0
Sept	0/70	1/204	2/93	0/35
Oct	0/117	8/234	2/57	0/0
Nov	0/151	5/206	4/150	0/0
Dec	4/139	9/163	6/118	0/0
Jan	2/130	14/197	7/91	0/0
Feb	3/134	8/172	5/99	1/17
Mar	0/124	6/207	0/69	0/21
Apr	0/128	1/219	0/52	0/21
May	0/130	0/246	0/75	0/30
June	0/110	0/262	0/90	0/021

* Minor poultry includes quail, pheasant, guinea fowl, pigeons are much lower in number and may not be available in the market for surveillance during some of the months.

## Presentation of the hypothesis

**Figure 1:**
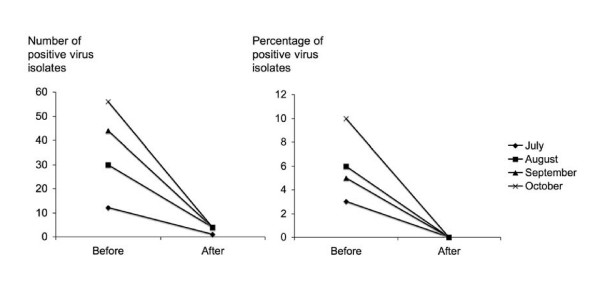
Comparison of H9N2 isolation rate before and after the rest day in 7 live poultry markets in Hong Kong (July to September, 2001). The reduction in isolation rates before and after the rest day in each month was statistically significant (p ≤ 0.01; Fisher's Exact test).

Poultry surveillance at the peak of the 1997 HKSAR epidemic showed that 20% of market poultry were infected by the virus.[7] The outbreak was controlled by the depopulation of all 1.5 million of poultry across the territory. No live ducks and geese were subsequently allowed to go into poultry markets because they are known to shed the virus without symptoms. Besides enforcing the accepted biosecurity measures in farms, a monthly moratorium or rest day with cleansing of all the poultry stalls were introduced to interrupt the transmission cycle in wet markets (Figure 1)[7]. In addition to biosecurity measures at the wet markets, vaccination of all farmed poultry and imported chickens was required to completely stop virus isolation in HKSAR. In Thailand, a strong geographical association was found between chicken outbreaks and free grazing ducks[8]. The Thai government has successfully mobilized and educated 1.2 million volunteers who reported on outbreaks in poultry which is one volunteer per ten families to look for early signs of bird flu in every village[9]. This huge team of public health volunteers has allowed the enforcement of a combination of measures which included early detection, culling poultry flocks, restricting poultry movement, and improving hygiene for the 1,417 affected villages in 60 of 76 provinces in 2004. These measures have brought the outbreak partially under control.

**Figure 2:**
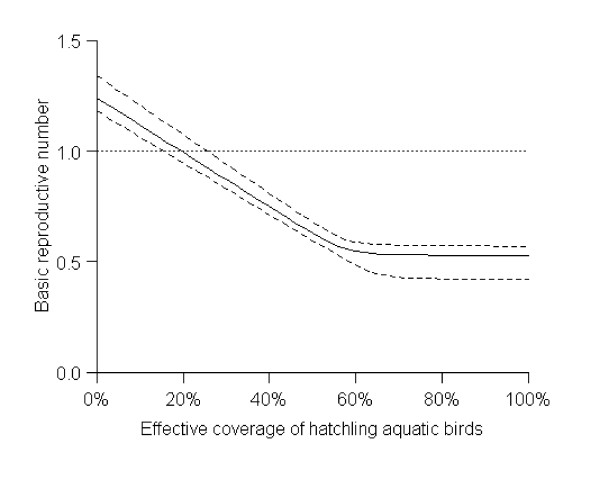
Impact of vaccinating aquatic birds (mainly ducks and geese) on the transmissibility of H5N1 influenza in poultry of a province in a South East Asian country. Maximum likelihood estimate (solid line) was made using isolation rates of 1.8% for aquatic birds and 0.26% for chicken [7] (*R*_0 _= 1.24, α_A _= 0.06 and *m *= 1.86). Dashed lines reflect a simple sensitivity analysis. We refitted the model to the highest and lowest isolation rates not significantly different from 1.8% and 0.26% (with 95% confidence). For simplicity, we assume aquatic birds and chickens mix freely, i.e. at any one time infections are not concentrated in only a few flocks. In this simple illustrative model, we did not account for the interaction between backyard flocks, industrial flocks, free ranging birds and wet markets. Note that these results are sensitive to the relative population sizes of the two groups (not shown). Therefore, further analysis of this system with a more detailed population structure is warranted.

As a proof of concept, we developed a simple mathematical model of coupled epidemics between aquatic birds (mainly ducks and geese) and chickens to estimate transmissibility within and between these two poultry populations. We used a susceptible-infected-recovered (SIR) structure for aquatic birds and susceptible-infected (SI) for chickens, assuming for simplicity that all chickens die after infection. The basic reproductive number, *R*_0_, is defined to be the expected number of infections generated by a single typically infectious aquatic bird or chicken in an otherwise susceptible population [10]. Preliminary results of the model show *R*_0 _= 1.14 and suggest that vaccinating ducks and geese would be a very efficient strategy to reduce *R*_0 _where coverage of 14% of hatchling aquatic birds, with no vaccination of chicken, might be sufficient to interrupt a self-sustaining outbreak, i.e. *R*_0 _< 1 (Figure 2)[11]. This does not necessarily mean that all transmission would be completely eliminated however in the absence of adjunctive measures such as environmental decontamination, control of importation of infected poultry, and chicken vaccination in industrial farms.

## Testing the hypothesis

### Why such control measures should be work

**Figure 3:**
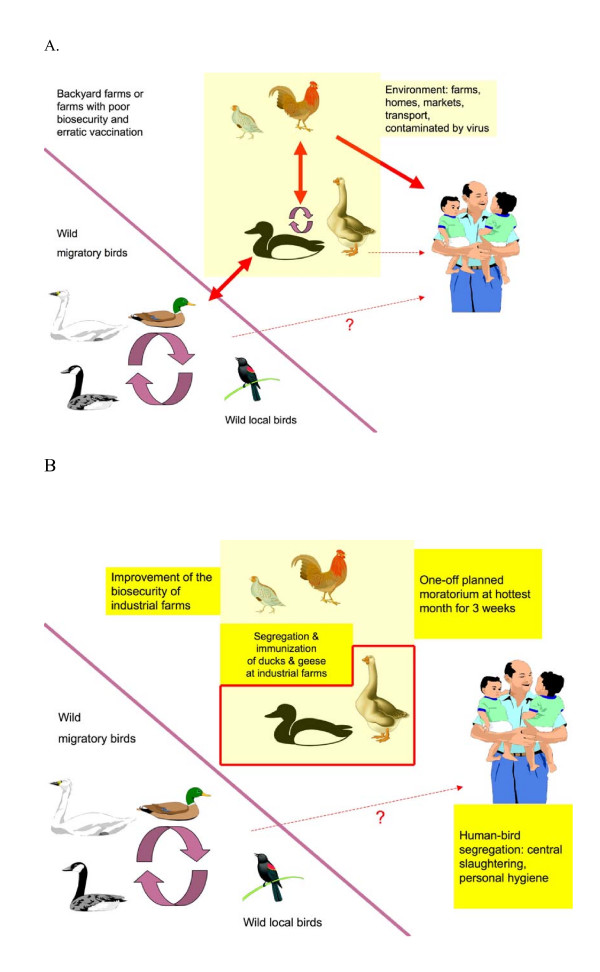
(A) Proposed transmission dynamics of influenza A H5N1 virus in wild birds and poultry. (B) Proposed control measures focusing on ducks and geese.

We propose to test these control measure in a province or a small district of any country highly endemic for influenza A H5N1 in their poultry (Figures 3a and 3b). Another province or district will be selected as the control where current disease control measures would be continued as recommended by the government.

### Selection of province or district

Although no major outbreak was observed recently, activity of H5N1 virus has been regularly detected from different types of apparently "healthy" poultry in past years. Thus the province or district selected should be the one which largely exports and not imports poultry.

### The team of officials and volunteers

Government officials, village chiefs, volunteers, and veterinarians are responsible for dissemination of information, patrolling the enforcement of moratorium, taking of specimens for virological surveillance, the repopulation of chicken and minor poultry in backyard farms and reporting of outbreaks in backyards. The provincial or district hospital superintendent, infection control team, microbiologists and clinicians are responsible for the clinical and virological surveillance in immunocompetent patients hospitalized for severe pneumonia.

### One-off poultry-free moratorium of 21 days

August is the hottest month with the highest environmental temperature which will spontaneously eliminate the virus shed into the environment. The government will announce the moratorium 4 months before the date so that all poultry are consumed or exported to other provinces by the end of July. This will minimize the economic loss of the farmers who can plan ahead for the moratorium.

### Poultry re-introduction

After three weeks of moratorium of poultry in the province or district, hatchlings of chickens and minor poultry will be re-introduced from qualified central supplies which can institute proper disinfection of the external surface of the fertilized poultry eggs for a restart of poultry farming. Mixed farming of chicken and duck and or geese with chickens and minor poultry will be permanently banned in the backyards. Geese and ducks will only be farmed in an industrial setting.

### Targeted immunization

Immunization of newly introduced poultry with standardized poultry vaccine will be implemented under the supervision of a dedicated team of veterinarians and local authority. A single type of vaccine with good track record should be used for the whole province [12]. Though a H5NX (X ≠ 1) vaccine is often recommended to facilitate subsequent serological surveillance to distinguish between natural seroconversion of poultry due to wild type infection or the result of vaccination, the use of an H5N1 vaccine antigenically matched to the circulating strain should also be considered to improve efficacy. The successful vaccination of duck and geese are confirmed by adequate haemagglutination inhibition titre before they are allowed to be freely grazing outside the pen.

### Law enforcement

Import of live poultry into the province will be banned but export of poultry will be permitted. All ducks, geese, are banned in backyards after the moratorium. Biosecurity of farms and markets are enforced to stop transmission cycles between wild birds and farm birds, between farms and between markets and farms. The supply chain of free virus-free chick seedlings for re-population will be a joint effort of the government and the industrial farms.

### Virological surveillance, isolation and characterization

Comprehensive surveillance of poultry in the test and control provinces or districts will be carried out to compare the outcome of the control measures over a period of spanning two winters. Cloacal, tracheal, and fecal samples were collected once every seven days from apparently healthy poultry in live-poultry markets. Virus isolates would be antigenically and genetically characterized as previously described [6].

All patients admitted to provincial or district hospitals for severe community-acquired pneumonia who are previously healthy, under the age of 65 with no underlying medical illness would be investigated for influenza A H5N1 virus infection in addition to the locally accepted routine investigations. Their throat swab and nasopharyngeal swab/aspirate are put into viral transport medium and sent to reference center for inoculation into MDCK cell line and chick embryo. Their acute and convalescent (14 days after onset of fever) sera would be tested for a four-fold rise in neutralizing antibody titer against the presently circulating genotype.

## Implications of the hypothesis

Even developed countries may not be able to withstand a rapidly spreading 1918-like influenza pandemic with a mortality rate of over 50%. Thus early intervention by quarantine of contacts, social distancing and rapid administration of a central stockpile of antiviral and vaccine to the targeted population at the epidemic centre appears to be a reasonable solution [13,14]. However it may be at least four weeks before cases of human-to-human transmission are reported to WHO due to the lack of infrastructure for epidemiological and virological surveillance in developing countries. Given the wide distribution of the endemicity of H5N1 virus in Asia, Middle East, Africa and Europe, it is not possible to predict the site to emerge a pandemic strain. Moreover, amantadine resistance was found in isolates from some affected areas and oseltamivir resistance have been detected in virus isolates before or after treatment [15-17]. There may not be adequate vaccine available and it is also possible that the stockpiled prototype vaccine may have a poor match to the pandemic strain. Despite the ability to rapidly scale up the vaccine production capacity and the use of potent immuno-adjuvant, at most only a third of the global human population may have the chance of getting the vaccine at least six months after the pandemic strain is identified. These almost insurmountable difficulties in pandemic preparedness and the present toll on poultry strongly suggest that primary attention should be turned to the control of the virus in the poultry while the virus has only limited capability for poultry-to- human transmission and before it changes to a pandemic strain. Until recently, depopulation based on clinical surveillance is the recommended field measure for the control of outbreaks. However such measures rely on the willingness of volunteers to report excess death especially in backyard poultry. Yet these volunteers may not necessarily be as willing to assist in enforcing more extreme preventive measures outside the settings of an impending outbreak, such as a moratorium or an embargo on raising geese and ducks in backyard flocks. Since these measures are associated with considerable losses to families involved, compensation must be given to the affected farmers in order to gain their cooperation and compliance to this intervention. Such extensive and prolonged efforts of surveillance in the field and laboratory will require technical, financial and administrative support from the developed countries, charity funds and international bodies.

Wild waterfowl including migratory ducks and geese are natural hosts of influenza A viruses. They could be asymptomatic despite infection by highly pathogenic H5N1 strains due to their unique immune system, or if they are previously exposed to other H5 viruses. Moreover highly pathogenic H5N1 strains can revert or be selected to lowly pathogenic strains when passaged in domestic ducks in the laboratory. Thus the duck may serve as the "Trojan horse" of H5N1 influenza viruses which continually to spread new strains to other poultry such the chicken wherein the virus may be selected to a highly pathogenic form[18,19]. The exchange of virus between migratory and domestic water fowls may have accounted for the spread of H5N1 infection over a vast geographic distance. Thus the water fowls especially the ducks and geese should be the focus for control. Many recent human cases were reported without concomitant findings of outbreaks in poultry. Our hypothesis if proven to work at a provincial level with poorly controlled outbreaks, can be adapted to smaller size districts without excess poultry deaths but virologically positive asymptomatic waterfowls. However this will mean a huge burden on laboratory surveillance since regular sampling of dead poultry in districts without excess poultry deaths have to be instituted.

Experience in the control of rinderpest in African cattle showed that it was difficult to achieve an adequate herd immunity by blanket vaccination, which may be wasteful or impractical [20]. Moreover moderate herd immunity with many asymptomatic virus shedders may actually help to sustain viral transmission. Therefore ducks and geese should only be reared in industrial farms where targeted vaccination programme can be fully implemented. The backyard virus is controlled by a one-off moratorium at the hottest month and re-populated by chickens and other minor poultry. As chickens and minor poultry are usually symptomatic if they have not been partially vaccinated or previously exposed to other H5 subtype viruses, they also serve as sentinel in monitoring the efficacy of control of H5N1 virus in ducks and geese. Vaccination programme in backyard farms is unlikely to be very effective unless the coverage is very comprehensive. It could be potentially detrimental since the vaccine giver may be carrying the virus from backyard to backyard. Partial immunity will only fuel the endemicity [20]. Therefore vaccination of chickens and minor poultry in backyards should be done if a good vaccination programme for ducks and geese is already in place.

Biosecurity measures at industrial farm and markets must be enforced to ensure that virus trafficking between those epidemic centres is minimized. For the wet markets of HKSAR, the ultimate goal is to go for central slaughtering with no more live chickens in retail markets. In order to decrease the resistance of the public and the poultry stall owners to the change, a series of transitional measures for prevention of avian influenza outbreaks have been implemented in HKSAR since 1997. Live ducks and geese are no longer allowed in the retail markets. Subsequently even live quails are not allowed since they can be the potential intermediate host for all subtypes of influenza viruses. Our local farmed and cross border imported chickens must be vaccinated against influenza virus H5 with sufficient serum HAI titre and negative cloacal swab by H5 RT-PCR before they are allowed into HKSAR or the wet markets. For local farms, biosecurity measures are tightened in terms of sufficient geographical separation and the use of bird-proof net. Chicken farms must not keep any other bird species and pigs. Movement of chickens or feed between farms is tightly controlled. Footwear is disinfected before entry to the farm. All staff must shower and put on protective clothing before entering the production area. Chickens from local farms should only be transported in disinfected cages to a designated local poultry wholesale market. Vehicles that carry chickens from one farm to the wholesale market are not permitted to carry chickens or any other poultry from another farm. No chickens that have been in a wholesale or retail market are allowed to enter local farms to stop a transmission cycle between the markets and the farms. All imported chickens must come from registered farms recognized by the government department. At the wholesale and retail market levels, market rest days (4 rest days for wholesale and 2 matching rest days for retail markets) are set up to break the virus replication cycle and reduce the viral load in the wet markets. During these rest days, all trading activities are stopped, all live poultry in the retail outlet slaughtered and the premises thoroughly cleansed and disinfected. With these comprehensive measures at farms and markets, the perimetric depopulation can be reserved for the control of any missed pockets of infection.

Unlike poultry, the role of wild birds in transmission disease to poultry or human is still poorly defined. Most would agree that the migratory birds are at least partly responsible for spreading the virus to poultry in widely separated geographical regions along their flyways. At least 10 dead birds were found to be infected with the H5N1 virus every winter in HKSAR. Local wild birds, migratory birds from the north and perhaps religiously released birds are implicated as the source of the virus in HKSAR (Personal communicaton by Richard Corlett). Though very few human cases have resulted from exposure to wild birds, the handling, slaughtering, defeathering and preparing of wild birds for consumption in endemic areas are considered as risk factors for acquisition of this disease. Due to outbreaks of disease in recreational park birds of HKSAR and their frequent contacts with human, the publics are advised to avoid contact with recreational park birds or wild birds. They should wash their hands with soap or disinfectant after contact. Though psittacine birds are generally more resistant to the H5N1 virus, pet birds caged in high concentration at pet bird shops should also be put under surveillance for outbreaks. As the religiously released birds are often wild birds caught in the mainland and smuggled into HKSAR, most of them will die after their release due to maladaptation to the new environment. Such activity should be banned on the ground of humanitarianism and public health. At the time of writing there is still no recommendation on the use of vaccine to prevent outbreaks in recreational park bird or pet birds due to the lack of understanding on the highly variable immune response of these highly diversified avian species to vaccination. In one study, some of these pet birds showed poor response to vaccination, whereas others such as the flamingos, ibis, rheas, Congo peafowl, black-winged stilts, Amazon parrots, and kookaburras showed good response to an inactivated H5N9 vaccine[21].

An effective poultry vaccine is one of the pillars in the proposed intervention. Between 1997 and 2001, HKSAR had failed to control outbreaks by relying on intensive clinical and virological surveillance of farms and markets with dedicated staff. Thus vaccination was used as a last resort in addition to other control measures. If a high compliance rate of vaccination can be achieved as in HKSAR, the risk of asymptomatic viral shedding is minimized. However, even among the domestic poultry, there are significant differences in their haemagglutination inhibition antibody response towards the commonly used inactivated H5 vaccines. Generally these vaccines has been found to be quite effective in chickens but the haemagglutination inhibition antibody titer tends to be lower in ducks and geese especially when given during the first few weeks of life when their immune system is relatively immature. Furthermore, H5N1 virus has become moreantigenicallydiversified in the last couple of years, it would be important to select antigenically matched or broadly cross reactive vaccines to maintain the efficacy of vaccination. A surveillance program should be established before and during the intervention to closely monitor the virological and serological results of vaccination. Similar to the human seasonal vaccine, a panel of vaccine candidates should be prepared and tested against many circulating strains before large scale production and field testing are done.

In summary, we proposed an intervention programme based on the successful experience of HKSAR in the control of the H5N1 virus by a combination of targeted immunization with the correct vaccine, segregation of poultry species and a three week moratorium of poultry farming (see additional file 1). This intervention, if successfully demonstrated at the provincial or district level in the developing country with persistent outbreaks, can be adapted at a much smaller scale for early intervention once the virus is found even if there is no excess death in the poultry population. But the later would require a strong laboratory support and regular sampling of all dead poultry. This may solve the present problem of human diseases without excess death in poultry.

## Competing interests

The author(s) declare that they have no competing interests.

## Authors' contributions

YKY, GY and CH initiated the study, reviewed the literature,  interpreted the data and wrote the manuscript.  YKY, GY, CH, LKS, RW, and JSMP jointly formulated the hypothesis. SR and  GML prepared the mathematical modeling and predictions. All authors  have read and approved the final manuscript.    

## Pre-publication history

The pre-publication history for this paper can be accessed here:



## Supplementary Material

Additional file 1 Mathematical model. Illustrate the potential efficacy of these interventions. Click here for file
