# Supplementary material for: A model to control the epidemic of H5N1 influenza at the source
Source: BMC Infect Dis. 2007 Nov 13;7:132. doi: 10.1186/1471-2334-7-132 (PMC2206044; doi:10.1186/1471-2334-7-132)
Supplement: Additional file 1 — Mathematical model. Illustrate the potential efficacy of these interventions. [file 1471-2334-7-132-S1.doc]

**Additional file: mathematical model**

In order to illustrate the potential efficacy of these interventions, we defined the following illustrative mathematical model. Let the size of the poultry and other aquatic bird population, relative to the size of the chicken population, be $N_{A}$. In this population; $I_{A}$ are infectious with H5N1, $R_{A}$ have been infected, have recovered and are now immune, and $S_{A}$ are susceptible. Similarly, let $I_{C}$ be the number of infectious poultry chicken and $S_{C}$ be the number of susceptible poultry chicken. We assume that all infected chicken die so there is no $R_{C}$ class. We let the hazard of infection for a single susceptible chicken at time $t$ be $\lambda_{C}(t)=\beta\left(I_{C}/N_{C}+m\alpha_{A}I_{A}/N_{A}\right)$, where; $\beta$ is the baseline transmissibility, $m$ is a symmetric coefficient mixing between the combined duck and geese flock and the chicken flock, $\alpha_{A}$ is the infectiousness of aquatic birds relative to chicken and $N_{A}$ is the size of the chicken population (set to 1 without loss of generality). Similarly, the hazard of infection for a single susceptible aquatic bird is $\lambda_{A}(t)=\beta\left(mI_{C}/N_{C}+I_{A}/N_{A}\right)$. A proportion $v$ of aquatic birds are successfully vaccinated at birth. The average duration of infectiousness of aquatic birds is $D_{A}$ and of chicken is $D_{C}$. The lifespan of aquatic birds is $L_{A}$ and of chicken is $L_{C}$. We balance deaths with births for both types of poultry which allows us to specify the model fully with three differential equations,

i.e. $R_{A}=N_{A}-S_{A}-I_{A}$ and $S_{C}=N_{C}-I$for all $t$.

Let the matrix

$\mathbf{M}=\left(\begin{matrix} (1-v)\alpha_{A}\left(\frac{1}{D_{A}}+\frac{1}{L_{A}}\right)^{-1} & (1-v)m\left(\frac{1}{D_{C}}+\frac{1}{L_{C}}\right)^{-1} \\ m\alpha_{A}\left(\frac{1}{D_{A}}+\frac{1}{L_{A}}\right)^{-1} & \left(\frac{1}{D_{C}}+\frac{1}{L_{C}}\right)^{-1} \end{matrix}\right)$.

We define the basic reproductive number$R_{0}=\beta\lambda$, where $\lambda$ is the largest eigenvalue of$\mathbf{M}$. The next generation matrix for the model is$\beta\mathbf{M}$. This definition is consistent with the basic reproductive number being the expected number of infections generated by a single typically infectious aquatic bird or chicken in an otherwise non-naturally infected population and allows us to parameterise the model in terms of $R_{0}$ rather than $\beta$. Note that we include the vaccination status of aquatic birds as an intrinsic property of the population. The “true” basic reproductive number, without consideration of vaccination, arises when$v=0$. Assumed parameter values are presented in Table S1.

**Table S1.** Assumed parameter values. The model is able to achieve a good fit to the two data points as long as there is not a substantial difference between $D_{DG}$ and $D_{C}$.

| Parameter | | Value | Notes / reference |
| --- | --- | --- | --- |
| $L_{A}$ | Lifespan of poultry geese and ducks | 49 days | Estimate, results not sensitive to this if substantially greater than $D_{DG}$ and $D_{C}$ |
| $L_{C}$ | Lifespan of poultry chicken | 49 days | Estimate, results not sensitive to this if substantially greater than $D_{DG}$ and $D_{C}$ |
| $N_{A}$ | Size of poultry geese and duck population | 146 million | 22 |
| $N_{C}$ | Size of chicken population | 374 million | 22 |
| $D_{A}$ | Duration of infectivity of geese and ducks with H5N1 | 4.5 days | 23 |
| $D_{C}$ | Duration of infectivity of chicken with H5N1 | 3.5 days | 24 |
